# Molecular and Morphological Characterization of *Eimeria crandallis* Isolated from Deer (*Cervidae*) in Different Captive Animals

**DOI:** 10.3390/life12101621

**Published:** 2022-10-17

**Authors:** Mian Abdul Hafeez, Adeel Sattar, Kiran Khalid, Abdur Rauf Khalid, Muhammad Shahid Mahmood, Muhammad Tahir Aleem, Kamran Ashraf, Faiza Aslam, Abdulaziz Alouffi, Aymen Mohammed, Mashal M. Almutairi, Muhammad Ikram ul Haq

**Affiliations:** 1 Department of Parasitology, University of Veterinary and Animal Sciences, Lahore 54000, Pakistan; 2 Department of Pharmacology and Toxicology, University of Veterinary and Animal Sciences, Lahore 54000, Pakistan; 3 Department of Livestock and Poultry Production, Faculty of Veterinary Sciences, Bahauddin Zakariya University, Multan 60800, Pakistan; 4 Institute of Microbiology, University of Agriculture, Faisalabad 38000, Pakistan; 5 MOE Joint International Research Laboratory of Animal Health and Food Safety, College of Veterinary Medicine, Nanjing Agricultural University, Nanjing 210095, China; 6 Department of Pathology, University of Veterinary and Animal Sciences, Lahore 54000, Pakistan; 7 King Abdulaziz City for Science and Technology, Riyadh 12354, Saudi Arabia; 8 Division of Molecular Therapeutics and Formulation, School of Pharmacy, University of Nottingham, Nottingham NG7 2RD, UK; 9 Department of Pharmacology and Toxicology, College of Pharmacy, King Saud University, Riyadh 11451, Saudi Arabia; 10 Veterinary Research Institute, Zarrar Shaheed Road, Lahore 54810, Pakistan

**Keywords:** coccidiosis, *Eimeria crandallis*, hog deer, Punjab urial, phylogenetic analysis, morphology

## Abstract

Coccidiosis is a protozoan disease that is characterized by diffuse diarrhea, dehydration, emaciation accompanied by moderate morbidity and mild mortality in animals and birds. The current study targeted the molecular characterization of *Eimeria* isolates in captive deer from different localities in Lahore. The host species was the *Cervidae* family, such as Hog deer (*Axis porcinus*) and Punjab urial (*Ovis aries vignei*). The *Eimeria crandallis* was isolated from zoo animals. The DNA was extracted from oocysts and amplified by using reported oligonucleotide primers that exhibited the 809 bp product. These were analyzed by using the small subunit 18S rRNA gene-based evolutionary relationship with 36 other *Eimeria* species reported in caprine, cervinae, bovines, avians, and rodents. Light microscopic examination exhibited 3.29% (7/213) *Eimeria*-positive fecal samples with morphological features, including sub-spherical forms, the presence of micropyle with polar cap, and oocysts diameters (μm) ranging from 24.32 ± 1.61 to 18.94 ± 1.51. The phylogenetic tree constitutes four distinct clusters with relatively higher values. The evolutionary network showed that sequences were clustered in the monophyletic group of *Eimeria* species reported in caprine and cervinae. The nucleotide and amino acid sequence similarity matrix analysis exhibited 99.5–99.9% identity of the study isolates with *Eimeria crandallis* (AF336339). This study provides relevant baseline data to develop strategic control measures for coccidiosis in zoo animals. However, further investigations are required to place the hog deer and Punjab urial-derived *E. crandallis* into the caprine-originated cluster.

## 1. Introduction

Coccidiosis is a gastrointestinal disorder caused by parasites of the genus *Eimeria*. The majority of the ruminants affected by this protozoan are juvenile and stressed animals [1]. It develops and propagates in the small and large intestines of animals. The Eimeria species is host-specific as it can affect sheep, birds, cattle, and many other wild animals [2]. Coccidiosis is a common disease among the wild ungulates contributing to high morbidity and mortality [3]. Coccidiosis may be asymptomatic or it may infest with profuse diarrhea, dehydration, depression, weakness, and poor appetite with other clinical symptoms depending upon the parasitic burden [4]. The incidence of coccidiosis on a farm may represent poor management and sanitation. On the other hand, coccidiosis in wild animals is less important than in domestic animals. However, overpopulation can lead to coccidiosis outbreaks with high mortality rates in young wild animals [5].

Different ruminant livestock species, including cattle, sheep, goats, and camelids, do not transmit *Eimeria* to one another. Currently, it is thought that transmission might occur between closely related wild and domestic animals [6,7]. There are reports of goat Eimeria species both in domestic and wild goats in Pakistan [8]. Moreover, wild sheep carrying the sheep *Eimeria* species include Bighorn sheep in North America and mouflons in Europe [9]. However, such statements are based upon the morphology of *Eimeria* oocysts, and species identification has yet to be confirmed by genetic studies or cross-infection trials. Therefore, it is still unclear if *Eimeria* species seen in wild animals are similar to those found in domestic animals or whether they are merely morphologically similar but different species.

*Eimeria crandallis* (*E. crandallis*) is considered a pathogenic species associated with weight loss. With respect to animals under stress, such as during transit, aggregation in new feedlots, or the initiation of dietary changes, a clinical-stage may evolve quickly [10]. *E. crandallis* are considered the most pathogenic because they can grow in Lieberkühn-crypt cells [11]. The oocysts of *E. crandallis* were observed in fecal samples of asymptomatic animals. *E. crandallis* is a substantial problem of ovine in different geographical regions of the globe [12]. Ovine coccidiosis can be a severe condition with negative economic effects: in asymptomatic animals, economic losses occur due to weight loss and, similarly, in symptomatic animals due to morbidity and mortality [13].

Although wild ungulates are an important part of the ecosystems they reside in, they are threatened in many parts of the world due to poaching, habitat loss, and competition with domestic cattle [14]. Hog deers (*Axis axis*) are mostly found in the grasslands of India, Nepal, Thailand, China, Vietnam, and Bangladesh, along rivers and mountains [15]. It is now extinct in many locations where it was formerly common, and its population has steadily declined [16]. In Pakistan, hog deers are restricted to the zoo, forests along rivers, grasslands, and particularly areas with thick grass and sparse plants [17].

Punjab urial (*Ovis vignei punjabiensis*) is a subspecies of Urial (*Ovis vignei*) that is only found in Pakistan. Their populations have declined by 30% due to hunting and poaching pressure [18]. These diminishing populations are threatened by anthropogenic and sympatric influences competing with one another, but diseases pose an even larger hazard [19]. One of the most common illnesses affecting wild ungulates and causing significant morbidity and death rates includes gastrointestinal infections. Few investigations have been conducted on diseases affecting urial populations [20].

In both wild and domestic ruminants, several *Eimeria* species may infect their host concurrently. There have been few studies concentrated on diseases that affect hog deer and Punjab urial populations. Studies on parasitic infestation such as coccidiosis are often focused on observation rather than genetic validations of the organisms detected. Microscopic testing takes time, and a small number of oocysts may not have the expected usual shape, rendering a proper diagnosis difficult. Additionally, there may be some overlap between biological traits, making it challenging in some circumstances to precisely identify an *Eimeria* species—something that can only be performed by trained experts [21]. Given the limits of microscopic diagnosis, genetic approaches have been developed to detect and specifically identify species of the genus *Eimeria* in various species such as poultry, ruminants, rabbits, and fishes [22].

Currently, no research has used molecular methods to diagnose *E. crandallis* infection in hog deer. Therefore, the current study aimed to investigate the presence of *E. crandallis* in hog deer found in different captive regions of Lahore, Pakistan. Attempts were also made to elucidate the evolutionary relationship of *E. crandallis* with other *Eimeria* species reported in avians, bovines, and rodents.

## 2. Materials and Methods

### 2.1. Study Area

The present research was carried out in various captive regions of district Lahore Pakistan. During the study period, the average temperature of Lahore was 29.84 °C (ranges from 25.5 °C to 33.9 °C). The annual rainfall was 607 mm, with a relative humidity of 60%.

### 2.2. Sample Collection

A total of 213 fecal samples of Hog deer (*Axis porcinus*) and Punjab urial (*Ovis aries vignei*) were collected from Lahore Zoo, Safari Park, and Jallo Park. The fecal samples were analyzed coprologically for the detection of *Eimeria* oocysts. The fecal samples were placed in plastic bags, properly labeled, and kept at a refrigerator temperature of 4 °C until coprological analysis.

### 2.3. Microscopic Examination

#### 2.3.1. Qualitative Examination

Fecal samples were analyzed qualitatively by flotation techniques using Sheather’s solution.

#### 2.3.2. Quantitative Examination

*Eimeria* oocyst-positive samples were subjected to quantitative evaluations by using the McMaster technique. After flotation, fecal sample was loaded on a McMaster chamber, and oocysts were counted in both chambers and multiplied by 50 to obtain the opg (oocyte per gram).

#### 2.3.3. Sporulation of Oocysts

Unsporulated oocysts were treated with 2.5% (*w*/*v*) potassium dichromate to induce sporulation. Oocysts were kept in 2.5% potassium dichromate for 48–72 h, and during this time, the sporocyst-to-oocysts ratio was verified.

#### 2.3.4. Morphological Characterization

The sporulated oocysts were examined at 400× magnification under a light microscope (Olympus, Tokyo, Japan) coupled with a digital camera. Fifty oocysts from each sample were randomly selected to observe their morphological characteristics. Identification was performed based on shape and size, the presence of micropyle, and polar caps [23].

### 2.4. DNA Extraction of Oocysts of E. crandallis

For the extraction of DNA, purified sporulated oocysts were centrifuged at 15,000× *g* for 3 min. The supernatant was discarded, and sediments were re-suspended in 20 μL sodium hypochlorite (7% wt/vol) at 4 °C for 2 h. The samples were mixed with 40 μL saturated salt solution following incubation at 55 °C for 1 h. The oocysts were allowed to homogenize in a TE buffer (300 μL), SDS (0.5%), and proteinase K (20 mg/mL). The rigid wall of sporulated oocysts was disrupted by speed sonication using the ultra-sonicator (Thomas Scientific, Swedesboro, NJ 08085, USA). The genomic DNA was extracted from excysted sporozoites using gDNA Mini kit Vizbio solutions (Korea) and analyzed by agarose gel electrophoresis [24].

#### Small Subunit 18S rRNA Gene Amplification and Polymerase Chain Reaction

**Figure 1:**
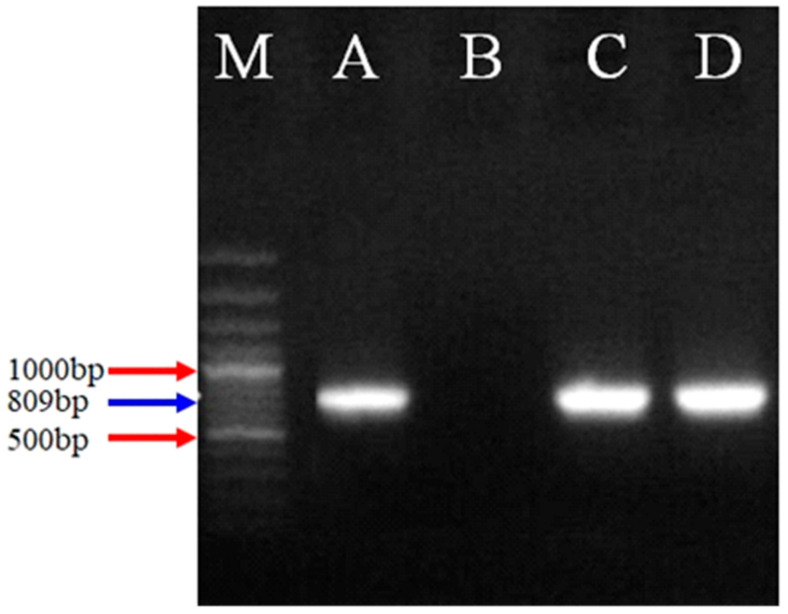
Ethidium bromide-stained agarose gel: Lane M with 100 bp DNA, Lane A possessing a positive control for the organism, Lane B with negative control (devoid of DNA), and Lane C and D indicate positive *E. crandallis* isolates with product size 809 bp (small subunit ribosomal RNA fragment).

The DNA from each sample was amplified by conventional PCR using previously reported oligonucleotide primer sequences with a predicted amplicon size of 809 bp (Figure 1); Forward5′-TATTTACGCAACTTCCCGACC-3′, Reverse 5′-AAGTATTCAGGGCGACAAGC-3′ [25]. The following PCR conditions were set on a T100 Thermal Cycler (Bio-Rad Laboratories, Hercules, CA, USA). Pre-denaturation was performed at 95 °C for 5 min followed by 33 cycles of each (denaturation (94 °C @ 30 s), annealing (54.5 °C @ 30 s), extension (72 °C @ 90 s) and a final extension (72 °C, 7 min). The PCR product was separated on 1.5% agarose gel containing 0.5 μg/mL ethidium bromide at 110 V, 230 mA for 30 min. in a gel electrophoresis chamber. Finally, the gel was visualized in a UV transilluminator.

### 2.5. Phylogenomic and Evolutionary Tree

For comparative evaluations, genome sequences of the rRNA gene were included with representatives of other organisms worldwide due to insufficient data available on *E. crandallis*. The retrieved nucleotide and deduced amino acid sequences were aligned by the ClustalW method (BioEdit^®^ version 7.2.5). The phylogenetic tree was constructed using the neighbor-joining model on MEGA X software. The reliability of the tree was based on 1000 bootstrap replicates, and the p-distance substitution model was applied [26]. Since the relationship among the populations does not conform to the tree-like pattern due to genetic polymorphism, partial rRNA genes were assessed for the evolutionary network. Amino acid multiple sequence alignment was evaluated on Web-Logo version 3.1 for comparative studies. The small subunit 18S rRNA gene-based evolutionary network was established in SplitsTree4 software by employing a neighbor-joining model [27].

### 2.6. Accession Numbers

The sequences are available in a public NCBI GenBank database under accession numbers MW449579.1 (*E. crandallis* isolate PU) and MW449580.1 (*E. crandallis* isolate HD).

### 2.7. Statistical Analysis

The prevalence rate was assessed by using te Z test. The Z value for this test was 2.7284, and the *p* value was 0.00634. These data showed a significant difference (*p* = 0.00634) between techniques used by using the Z Test.

## 3. Results

### 3.1. Detection and Morphological Characteristics of E. crandallis Oocysts

**Figure 2:**
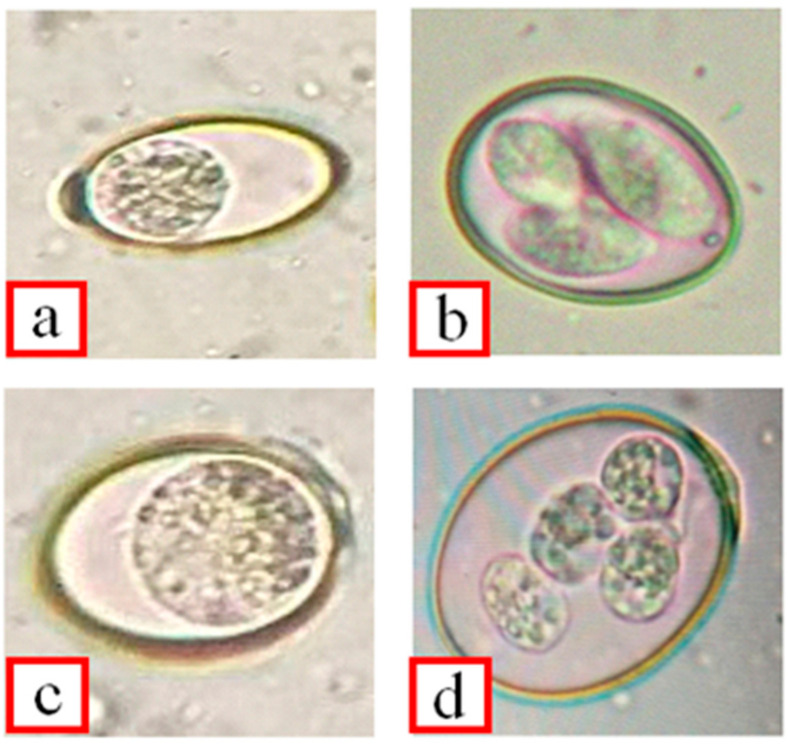
Oocysts were diagnosed in this study: unsporulated (**a**), sporulated (**b**) oocyst from HD, unsporulated (**c**), sporulated, and (**d**) oocyst from PU.

Out of all fecal samples examined, 3.29% (7/213) scored positive for *E. crandallis* oocysts. The positivity of *Eimeria* oocysts in Lahore Zoo, Safari Park, and Jallo Park is described in (Table 1). Data detecting the morphological features include sub-spherical forms, the presence of micropyle with polar cap (Figure 2), oocysts diameters (μm) ranging from 24.32 ± 1.61 to 18.94 ± 1.51, and sporocyst diameters (μm) ranging from 10.68 ± 1.39 to 7.29 ± 0.84. Un-sporulated oocysts had a broad ellipsoidal to spherical shape with a smooth wall and had yellow coloring; the micropyle was, however, presented with polar caps. Sporulated oocysts had an ellipsoidal shape with four sporocysts, and each sporocyst had two sporozoites.

### 3.2. Molecular Detection and Phylogenetic Analysis of E. crandalis Oocysts

**Figure 3:**
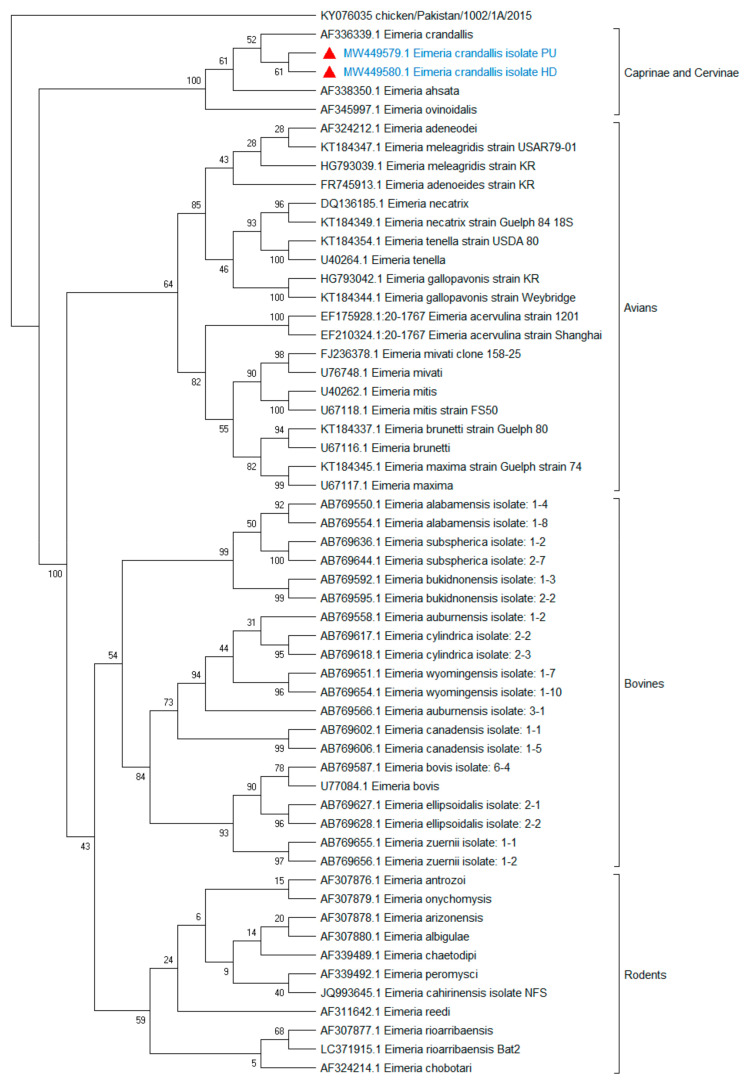
Small subunit 18S rRNA gene-based phylogenetic analysis of Eimeria isolates originated from caprine, bovine, avian, and rodents. The evolutionary tree was generated with MEGA X software using the neighbor-joining method with a reliability of 1000 bootstrap intervals. Red triangles revealed current isolates (*Eimeria crandallis*).

**Figure 4:**
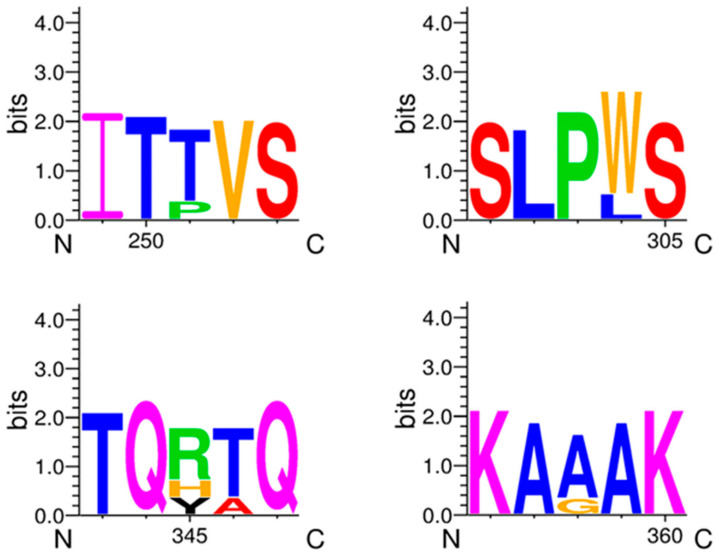
Comparative analysis of amino acid substitutions in caprine-originated Eimeria species. Multiple alignments and amino acid variations at each position were determined using WebLogo 3.1.

**Figure 5:**
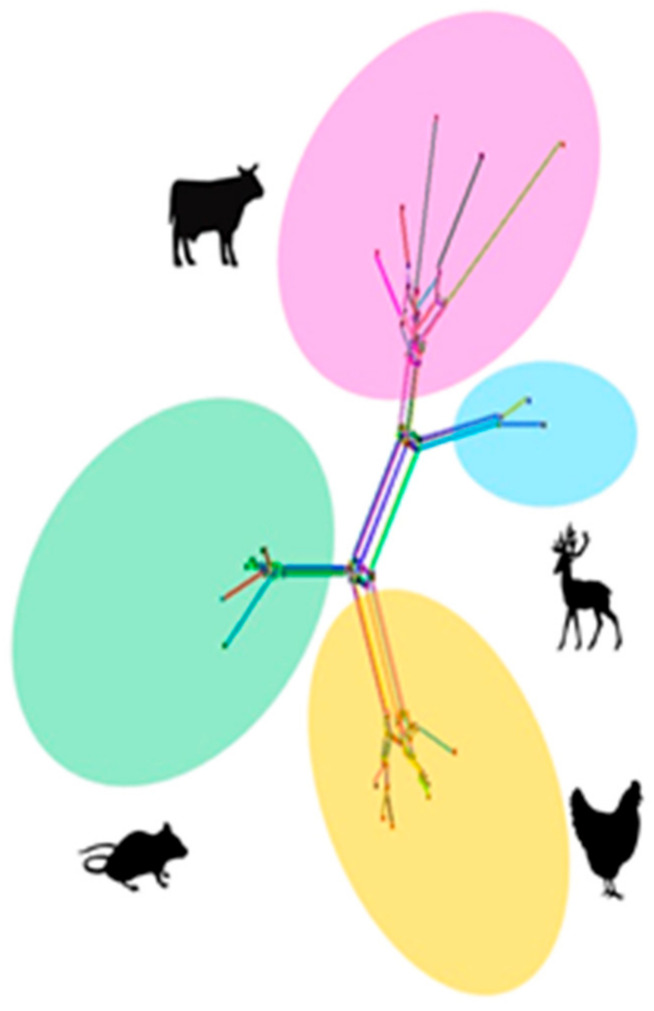
Evolutionary network based on the Neighbour-net, including sequences of Eimeria, (purple, blue, green, and orange circles indicating a clade representing Eimeria sequences of the specific host). The Splits Tree software was employed to generate the evolutionary network based on an uncorrected p-distance, using the Kimura 2-parameter substitution model.

In spite of seven microscopically detected *Eimeria* oocyst fecal samples, only two samples amplified the small subunit 18S rRNA gene, remaining exhibited no amplifications. For phylogenetic analyses, small subunit 18S rRNA gene sequences of *E. crandallis* (MW449580, MW449579, and AF336339) and 33 other species of *Eimeria* from caprine and cervine, bovines, avian, and rodents were employed. The evolutionary tree (Figure 3) indicated that *E. crandallis* isolated from hog deer (MW449580) and Punjab urial (MW449579) were grouped in a clade consisting of *E. crandallis* (AF336339), *E. ahsata* (AF338350), and *E. ovinoidalis* (AF345997) (Figure 3). The detailed information of *Eimeria* species sequences employed to determine the nucleotide and amino acid homology matrix is shown in Table 2. This analysis revealed that *E. crandallis* in the current study exhibited 99.5–99.9% nucleotide identity with *E. crandallis* (AF336339), *E. ahsata* (AF338350), and *E. ovinoidalis* (AF345997). The nucleotide sequence alignment of caprine and cervine originating from fives isolates revealed that threonine (position: 251, 346), tryptophan (position: 304), arginine (position: 345), and alanine (position: 358) are substituted by proline, leucine, histidine, tyrosine, and glycine, respectively (Figure 4). In addition, the evolutionary network generated by Splits Tree revealed the distinctive phylogenetic relationship between the *Eimeria* species isolated from caprine, avian, bovine, and rodents (Figure 5). This analysis also demonstrated the evolutionary dynamics of *Eimeria* species based on small subunit 18S rRNA genes.

## 4. Discussion

Coccidiosis is an important disease in wild animals caused by *Eimeria* spp. *Eimeria* oocysts are secreted in the feces after multiplying in their host’s intestinal cells, spreading in the environment around animals. The fecal–oral pathway causes infection in other animals who share the living space. For instance, oocysts are often detected in the ewe’s udder and the litter, which raises the possibility of infection [41]. Ingested parasite loads, *Eimeria species*, other diseases present, age, the immunocompetent state of the host, and farming methods are only a few causes that might affect the development of clinical coccidiosis [42].

*E. crandallis* has the ability to induce clinical infestations along with *E. ovinoidulis.* However, it may persist in the herds without showing any clinical signs. The current study revealed that *E. crandallis* parasitized the nucleus of host intestinal epithelial cells and ultimately divides them by cell synchronization [10].

The morphological characteristics of *E. crandallis* include a sub-spherical form accompanied by the presence of a micropyle and polar cap. The oocyst’s diameter was reported to range from 24.79 ± 1.72 to 19.1 ± 1.46 µm, while the sporocyst’s diameter (μm) ranged from 10.56 ± 1.46 to 7.08 ± 0.79 [43]. The identification of the *Eimeria* spp. was conducted by measurements of the diameter of non-sporulated and sporulated oocysts, their shape and color, sporocyst size, and the presence or absence of micropyles [44]. Similarly to our results, [45] stated that the size of the oocyst was 414.2 × 11.7 μm, and the size of sporocysts was 7.6 × 4.2 μm. The authors of [46] studied the morphological parameters of *Eimeria* spp. of sheep and revealed that morphometric characteristics differentiate them. In this study, the morphology of *E. crandallis* showed oocyst and sporocyst diameters at 26.46 ± 1.74 and 19.56 ± 1.34, respectively.

These results are in agreement with current findings. A higher infection rate (6.67%) of *Eimeria* spp. was reported in China in captive forest musk deers [47] and the Central Iberian Peninsula, at 13.3% [48]. These results are in line with the findings of [49], which reported 4.1% of red deer in Spain. However, a lower prevalence was also reported in Poland, at 1.6% [50]. The molecular occurrence of *E. crandallis* in sheep was 30.38% [51]. The investigation of *Eimeria* spp. in deer using molecular techniques reflected a higher infection rate of 77% [52]. The prevalence of *E. crandallis* in sheep was 40.2% in Dakahlia, Egypt [10]. The prevalence of *E. crandallis* differs significantly in different areas due to differences in weather, the type of management, hygiene, the method of feeding, weaning, and the presence of other infections [53]. Variations in prevalence may be caused by a variety of elements, such as the environment, stocking density, and the possibility of feed/water pollution.

Previously, the prevalence of Eimeria in deer in different areas of the world was studied. In China, forest musk deer had a 65% incidence of Eimeria [54]. The prevalence of *Eimeria* in semi-domesticated reindeer (*Rangifer tarandus wardi*) in Norway was 23% overall [55]. *Eimeria* spp. were detected in moose (*Alces alces*) in Poland at a rate of 1.6 to 4.3% [56]. The incidence of *Eimeria* was found to be 9% in a confined herd of caribou (*Rangifer tarandus caribou*) in Canada [57]. Moose (*Alces alces*) and mule deer (*Odocoileus hemionus*) have substantially lower prevalence rates of 1.6% in Poland [50] and 2% in the US, respectively [58]. Sika deer (*Cervus nippon*) had a 14.8% prevalence rate in Austria [59].

Concerning the molecular typing of *Eimeria* based on subunit 18S rRNA gene, the phylogram indicates that field isolates of *E. crandallis* exhibited a close relationship with the already reported *E. crandallis*, *E. ahsatam* and *E. ovinoidalis*; hence, it is placed in a monophyletic group. The nucleotide and amino acid homology index represents more than 99% identity with these isolates. Current findings are in accordance with those reported by other scientists, who kept *E. crandallis*, *E. ahsata*, and *E. ovinoidalis* in a single group based on 18S rRNA sequence analyses, despite many pathological and biological features differences [12]. Similarly, in another study, they also placed sheep *Eimeria* spp. in a single group, separating it from large ruminants [29]. In contrast to our findings, Trejo-Huitrón et al. placed sheep *Eimeria* spp. into two clades [46]. This difference may be caused by the fact that five species were taken into account for this study. Two species used in this study have a difference in morphological characteristics, i.e., the presence and absence of residual body. The ITS1 region has a low intraspecific and substantial interspecific variance, preventing cross-identifications between genus species [21]. Moreover, it has been reported that *Eimeria* species originated from avians, bovines, and rodents and tend to form separate clades in phylogram [60]. Hence, they can be used for their identification in environmental samples.

## 5. Conclusions

In conclusion, the current study revealed the molecular and morphological prevalence of *E. crandallis* in hog deer and Punjab urial present in different captive regions of the Lahore district, Pakistan. This study provides relevant baseline data to develop strategic control measures for coccidiosis. For future prospects, attention can be focused on molecular conformation and the characterization of *Eimeria* species in zoo animals.

## Figures and Tables

**Table 1:** Positivity for *E. crandallis* in hog deer and Punjab urial in different captive locations.

Site	Hog Deer % (n/N)	Punjab Urial % (n/N)
Lahore zoo	8.51% (4/47)	7.69% (3/39)
Jallo Park	0.00% (0/31)	0.00% (0/24)
Safari Park	0.00% (0/41)	0.00% (0/31)

**Table 2:** Detailed information of Eimeria sequences to determine percentage identity. The nucleotide and amino acid homology matrix were evaluated using the DNASTAR Lasergene MegAlign version 7.1.0 (44) tool.

Accession No.	Organism	Host	Country	Collection Year	Reference
MW449580	*E. crandallis* isolate HD	Hog Deer	Pakistan	2019	This study
MW449579	*E. crandallis* isolate PU	Punjab Urial	Pakistan	2019	This study
AF336339	*E. crandallis*	Sheep	Turkey	2001	[28]
AF338350	*E. ahsata*	Sheep	Turkey	2001	[28]
AF345997	*E. ovinoidalis*	Sheep	Turkey	2001	[28]
MF356556	*E. arloingi*	Capra aegagrus hircus	Portugal	2012	[29]
AB769554	*E. alabamensis*	Bos taurus	Japan	2013	[30]
AB769558	*E. auburnensis*	Bos taurus	Japan	2013	[30]
AB769587	*E. bovis*	Bos taurus	Japan	2013	[30]
AB769592	*E. bukidnonensis*	Bos taurus	Japan	2013	[30]
AB769602	*E. Canadensis*	Bos taurus	Japan	2013	[30]
AB769618	*E. cylindrical*	Bos taurus	Japan	2013	[30]
AB769628	*E. ellipsoidalis*	Bos taurus	Japan	2013	[30]
AB769636	*E. subspherical*	Bos taurus	Japan	2013	[30]
AB769654	*E. wyomingensis*	Bos taurus	Japan	2013	[30]
AB769656	*E. zuernii*	Bos taurus	Japan	2013	[30]
EF210324	*E. acervuline*	Gallus gallus	China	2006	[31]
AF324212	*E. adeneodei*	*Meleagris* (turkey)	USA	2001	[32]
U67116	*E. brunette*	Gallus gallus	USA	1997	[33]
HG793042	*E. gallopavonis*	Turkey	Czech Republic	2013	[34]
U67117	*E. maxima*	Gallus gallus	USA	1997	[33]
KT184347	*E. meleagridis*	Meleagris gallopavo	USA	2011	[35]
U67118	*E. mitis*	Gallus gallus	USA	1997	[33]
U76748	*E. mivati*	Gallus gallus	USA	1997	[33]
DQ136185	*E. necatrix*	Gallus gallus	China	2005	[36]
U40264	*E. tenella*	Gallus gallus	USA	1995	[37]
AF307880	*E. albigulae*	Wood rat	USA	2000	[38]
AF307876	*E. antrozoi*	Rodents	USA	2000	[38]
AF307878	*E. arizonensis*	Deer mouse	USA	2000	[38]
JQ993645	*E. cahirinensis*	Acomys dimidiatus	Czech Republic	2012	[32]
AF339489	*E. chaetodipi*	Pocket mouse	USA	2001	[39]
AF324214	*E. chobotari*	Kangaroo rat	USA	2000	[32]
AF307879	*E. onychomysis*	Grasshopper mouse	USA	2000	[38]
AF339492	*E. peromysci*	Deer mouse	USA	2001	[39]
AF311642	*E. reedi*	Rodents	USA	2000	[40]
AF307877	*E. rioarribaensis*	Rodents	USA	2000	[38]
